# Genetic Variants from Large Cohorts and Familial Studies Implicate Common Mechanisms in Schizophrenia

**DOI:** 10.3390/biology15070531

**Published:** 2026-03-26

**Authors:** Ambreen Kanwal, José V. Pardo, Sadaf Naz

**Affiliations:** 1 Department of Genetics, Washington University in Saint Louis, Saint Louis, MO 63104, USA; 2 Department of Psychiatry, University of Minnesota, Minneapolis, MN 55455, USA; jvpardo@umn.edu; 3 Minneapolis Veterans Affairs Health Care System, Minneapolis, MN 55417, USA; 4 School of Biological Sciences, University of the Punjab, Lahore 54590, Pakistan

**Keywords:** chromatin modification, glutamatergic system, psychosis

## Abstract

Schizophrenia has an estimated population prevalence of 1%, but the etiology of this devastating psychiatric condition remains largely uncharacterized. A pronounced genetic component underlies schizophrenia, with heritability estimates ranging from 60% to 80%. Until now, genome-wide association studies have successfully identified 287 distinct genetic loci associated with schizophrenia, but these primarily involve common variants that have minimal individual risk. The recent advent of exome sequencing and genome sequencing has identified ultra-rare sequence variants associated with schizophrenia in familial cases as well as in large cohorts. These studies have implicated multiple gene variants that individually have a large effect size in contributing to schizophrenia. A comparison indicates that these genes exhibit high expression levels in the central nervous system and their protein products participate in many converging pathways encompassing synaptic transmission, glutamatergic neurotransmission, chromatin modification processes, transcriptional regulation, and ubiquitin–proteasome degradation. Although model systems have been established for some genes, most remain to be further studied to identify how gene dysfunction correlates with disease.

## Simple Summary

Schizophrenia is a devastating mental illness with high heritability ranging from 60% to 80%. Multiple genetic studies identified both common and rare variants associated with this debilitating brain disorder. These studies implicated genes involved in brain functions that mostly converge on synaptic transmission, glutamatergic pathways, chromatin modification, transcriptional regulation, and the ubiquitin–proteasome pathway. Functional studies for a few genes have been carried out, and the underlying mechanisms of pathogenesis for the others remain to be elucidated.

## 1. Introduction

Schizophrenia is a profoundly debilitating psychiatric disorder that presents a complex constellation of symptoms, broadly categorized as positive, negative, cognitive, and affective. Positive symptoms are characterized by the presence of hallucinations (which may be visual, auditory, or tactile) and delusional beliefs, while negative symptoms include antisocial conduct, catatonia, a diminished capacity for emotional expression, alogia, anhedonia, speech impediments, and blunted affect [1]. Affective symptoms encompass suicidal ideation, disordered thinking, disorganized behavior, and anxiety, whereas some cognitive symptoms associated with schizophrenia include functional deficits, difficulty focusing attention, challenges with decision-making, organizing, learning, and recalling information [2].

About 0.4–1% of people are reported to be affected with schizophrenia worldwide [3,4]. Schizophrenia is a complex disorder with an interplay between environmental exposures interacting with genetic factors contributing to risk for this devastating mental illness [5]. Epidemiological studies indicate that maternal stress, particularly during the first and second trimesters, can alter gene expression, making the fetus comparatively more prone to develop schizophrenia spectrum disorders [6]. Early environmental factors that may significantly contribute to the development of schizophrenia include maternal stress, prenatal malnutrition, obstetric complications, and adverse childhood experiences (ACEs) [6]. In contrast, migration, urbanicity, and use of cannabis are later-life exposures that have been studied as contributing factors for schizophrenia [7]. Family, twin, and adoption studies have revealed high heritability of schizophrenia, with a range between 60 and 80% [8,9].

We review the genetic landscape of schizophrenia as delineated by genome-wide association studies (GWAS), as well as research that implicates rare variants with high disease impact in mixed cohorts and familial cases of schizophrenia. Despite the complexity of the disorder and the extensive findings on the genetics, the existing evidence points to converging pathways such as glutamatergic signaling, synaptic function, and neurotransmission, which, when affected, underlie schizophrenia.

## 2. Materials and Methods

We performed a narrative review to investigate the genetic landscape of schizophrenia. We used the “advanced literature search” feature in PubMed without time filtering up to February 2026. We queried the database using keywords such as “schizophrenia heritability”; “schizophrenia and genes”; schizophrenia and GWAS”; “schizophrenia and CNV”; “schizophrenia and rare variants”; “schizophrenia and exome”. We selected genetic studies from very large cohorts or families, including GWAS, rare-variant analysis, de novo variants, and copy-number variants. We reviewed the abstracts of the listed publications and shortlisted all those that reported genes associated with the disorder. We excluded studies that yielded no positive findings, identified genes without expression in the nervous system, and failed to replicate previous findings.

Similarly, we also searched the Online Mendelian Inheritance in Man database (OMIM; https://www.omim.org/; accessed 1 February 2026) using the search term “schizophrenia and genes.” We included molecular genetic studies that identified genes or loci that segregate with the disease phenotype. However, we excluded studies of genes identified through gene expression analysis, such as microarray or transcriptomic analysis.

Once all the publications were shortlisted, we read the manuscripts and synthesized the review. We separated the gene studies by the method used for identification for tabulation. Next, we reviewed the information and identified genes based on the implicated pathways.

## 3. Heritability and Genetic Factors

A meta-analysis investigating the heritability of psychotic disorders indicated a substantial genetic component in the etiology of schizophrenia; the proband monozygotic and dizygotic concordance rates were 33% and 7%, respectively [10]. The proband concordance rate is the probability of developing a trait if the co-twin also has that trait. Familial studies suggest that individuals with affected first-degree relatives are 6.4 times more likely to develop schizophrenia, while those with affected second-degree relatives have a 2.4 times higher risk compared to the general population [4]. Furthermore, children with one affected parent face a 13% risk, and those with two affected parents have a 50% risk of developing schizophrenia [11].

## 4. Early Studies to Identify Susceptibility Genes for Schizophrenia

The first systematic study of the genetics of a psychiatric disorder was performed by Ernst Rüdin in 1916. Rüdin conducted a family-based study and found that brain disorders did not fit a simple Mendelian, monogenic mode of transmission [12]. He proposed multiple models, including oligogenic inheritance, incomplete penetrance, and a two-locus recessive model [12]. Numerous familial investigations continued to delineate the genetic basis of schizophrenia. Early on, dopaminergic dysregulation was considered the main underlying pathway for schizophrenia, and most studies were carried out to evaluate the role of *DRD2* genetic variants in multiplex families. However, this research did not support the hypothesis that *DRD2* variants were involved [13,14,15]. A few other studies genotyped variants in different genes, such as *MTHFR* [16,17], *CHI3L1* [18], *DISC1* and *DISC2* [19], *SYN1* [20], *DRD3* [21] and *RTN4R* [22] and linked them to increased susceptibility to schizophrenia. A number of cytogenetic locations, such as 5q31.1-q35.1 [23], 6p22.1-p21.3 [24], 6q25 [25], 8p22-p21 and 13q32 [26], 10q22.3 [27] and 18p [28] were identified as risk loci for schizophrenia. It is interesting to note that most of these loci were identified through genome-wide linkage disequilibrium (LD) studies in multiplex families. Other research revealed an increased risk of developing schizophrenia (around tenfold) in the offspring of an affected individual compared to the general population [15]. Multiple twin studies were also performed in identical (monozygotic) and fraternal (dizygotic) twins, which revealed a 40–50% concordance rate of schizophrenia in identical twins [29,30]. Adoption studies were carried out to test the environmental contributions to schizophrenia. However, genetics was found to play a more significant role than the environment or family experiences. Mental disorders were found to be more common in children whose biological parents had schizophrenia, regardless of whether they were raised by healthy or schizophrenic adoptive parents. Conversely, children adopted by schizophrenic parents, who were born to healthy biological parents, did not show an increased risk of developing mental disorders [31,32]. Thus, these findings demonstrated a strong genetic component for schizophrenia and laid the foundation for future genome-wide association analyses and familial studies for identifying the loci associated with schizophrenia.

## 5. GWAS for Schizophrenia

Genome-wide association studies (GWAS) have identified numerous loci associated with schizophrenia over the past two decades [33]. One of the first GWAS reported three loci with the strongest independent association around *ZNF804A* to schizophrenia in 479 sporadic cases versus 2937 controls [34] (Table 1). Case–control association studies in Ashkenazi ethnicity also implicated genes in the glutamatergic system in psychosis [35]. Subsequent studies with larger sample sizes identified additional associated loci. A GWAS of 108 potential loci in 36,989 patients versus 113,075 controls revealed single-nucleotide polymorphisms (SNPs) in seven genes significantly associated with schizophrenia: *DRD2*, *GRM3*, *GRIN2A*, *SRR, GRIA1*, *CACNA1C, CACNB2,* and *CACNA1I* [36] (Table 1). Other large-scale collaborative GWAS increased the number of loci associated with schizophrenia to 287 in over 76,755 cases vs. 243,649 controls. Among these loci, 120 genes were prioritized, of which seven showed credible causal variants. Of these, rare and common disruptive coding variants in the genes *GRIN2A*, *SP4*, *STAG1*, and *FAM120A* were strongly associated with schizophrenia [8] (Table 1).

As each GWAS-identified variant had a high population allele frequency and conferred a relatively small individual risk for schizophrenia, these were referred to as common variants [38]. Notably, GWAS have identified thousands of common alleles collectively conferring schizophrenia risk; however, none achieved significance on an individual level [38]. Secondly, the association between common SNPs and schizophrenia found by GWAS does not necessarily implicate causal pathways. These potential relationships may be interpreted as correlations. For example, a common SNP may be associated with increased risk, but this could be due to linkage with other causative factors or to its indirect influence on a biological pathway. Thus, definitive information regarding how these common variants lead to the symptoms of schizophrenia is often lacking. Therefore, multiple studies aimed at identifying rare deleterious variants have also been conducted.

## 6. Massively Parallel Sequencing Studies for Large Cohorts of Schizophrenia

While schizophrenia is generally accepted to be a complex polygenic disorder arising from common variants in multiple genes, rare, high-impact variants also contribute to its manifestation [39,40]. A comprehensive whole-genome sequencing study involving 112 patients with extreme early-onset, treatment-resistant schizophrenia and 4185 controls identified a significantly higher prevalence of rare, damaging missense variants in genes such as *ACACA*, *CACNA1C*, and *GABRA* among the schizophrenia patients [41] (Table 2). Specifically, 48.2% of treatment-resistant schizophrenia patients had at least one damaging missense or loss-of-function variant in a variant-intolerant gene, compared to only 25.4% in controls. Markedly, loss-of-function variants were significantly enriched in genes associated with Mendelian syndromes characterized by aggressive behaviors or hallucinations [41].

In the exploration of ultra-rare variants conferring substantial risk for schizophrenia, exome sequencing of 24,248 patients and 97,322 controls revealed ten genes [42] (Table 2). These play roles in the glutamatergic system, histone modification, and the ubiquitin–proteasome system, which are thought to be disrupted by variants in these genes [43]. Similarly, ultra-rare pathogenic variants in a cadherin-related gene [44] involved in neuronal signaling further supported the contribution of rare variants as a major risk in a subset of schizophrenia cases [45] (Table 2). More recently, exome sequencing was performed for 4650 patients versus 5719 controls. The findings were combined with previously published sequencing data, resulting in an aggregate of 28,898 cases, 103,041 controls, and 3444 proband–parent trios. Rare genetic variants were found in eight genes, suggesting these genes as novel risk genes for schizophrenia [46] (Table 2).

**Table 2:** Summary of genes with rare variants implicated in schizophrenia pathogenesis through massively parallel sequencing in large cohorts.

Gene Symbol	Gene Name	Putative Role in Schizophrenia Pathogenesis	Mechanism Involved	References
*ACACA*	Acetyl-CoA carboxylase alpha	Encodes a cytosolic enzyme that plays a critical role in lipid metabolism	Neuronal differentiation	[41]
*AKAP11*	A-kinase anchoring protein 11	Is a target of E3 ubiquitin ligases	Ubiquitin–proteasome system impairment	[42]
*BSCL2*	Lipid droplet biogenesis-associated seipin	Forms lipid droplets and plays a role in lipid metabolism	Neuronal differentiation	[46]
*CACNA1C*	Voltage-dependent L-type calcium channel subunit alpha-1C	The pore-forming alpha-1C subunit of the voltage-gated calcium channel is responsible for generating L-type calcium currents	Calcium signaling and neuronal activity	[41]
*CACNA1G*	Calcium voltage-gated channel subunit alpha1 G	Encodes the α1G subunit of the T-type calcium channels	Calcium signaling and neuronal activity	[42]
*CGREF1*	Cell growth regulator with EF-hand domain 1	Encodes a secreted protein that acts as a negative regulator of cell proliferation by modulating key transcriptional and signaling pathways	Neural functions	[46]
*CUL1*	Cullin 1	Encodes a component of an E3 ubiquitin ligase complex, an enzyme involved in protein modification	Ubiquitin–proteasome system impairment	[42]
*GABRA*	Gamma-aminobutyric acid (GABA) A receptor, alpha subunit	Forms a receptor that responds to the neurotransmitter GABA, which is the primary inhibitory neurotransmitter in the CNS	GABAergic pathway	[41]
*GRIA3*	Glutamate ionotropic receptor AMPA type subunit 3	Involved in ionotropic glutamatergic transmission	Component of the glutamatergic system	[42]
*GRIN2A*	Glutamate receptor ionotropic N-methyl-D-aspartate subunit 2A	Encodes a NMDAR that increases calcium influx in neurons and regulates neuronal signaling	Component of the glutamatergic system	[42]
*HERC1*	HECT and RLD domain-containing E3 ubiquitin protein ligase family member 1	Encodes an E3 ubiquitin ligase and targets specific proteins for degradation	Ubiquitin–proteasome system impairment	[42]
*KLC1*	Kinesin light chain 1	Encodes a light chain subunit of kinesin, which is a tetrameric protein complex that facilitates intracellular transport along the cytoskeletal framework	Neuronal differentiation	[46,47]
*OTUD7A*	OTU domain-containing protein 7A	Deubiquitinase, which cleaves ‘Lys-11’-linked polyubiquitin chains from proteins, affecting their stability and function	Ubiquitin–proteasome system impairment	[45]
*PCDHA3*	Protocadherin alpha 3	It is part of the cadherin superfamily and is known to mediate homophilic cell adhesion while maintaining neuronal connections	Neuronal synaptic connections	[44]
*PCLO*	Piccolo presynaptic cytomatrix protein	Encodes a component of the presynaptic active zone in neurons, which plays a crucial role in regulating synaptic vesicle exocytosis	Neurotransmission	[46]
*RB1CC1*	RB1 inducible coiled-coil 1	Encodes a DNA-binding transcription factor that recognizes GC-rich sequences around the promoters of various genes and activates transcription	Autophagy and neuronal migration	[42]
*SETD1A*	SET domain containing 1A histone lysine methyltransferase	Regulates gene expression	Chromatin remodeling	[42]
*SLC6A1*	Solute carrier family 6 member 1	Encodes a gamma-aminobutyric acid (GABA) transporter, which facilitates the reuptake of GABA from the synaptic cleft of inhibitory synapses	GABAergic pathway	[46]
*SP4*	Sp4 transcription factor	Encodes a transcription factor that interacts with the promoter region of various genes and modulates their expression; regulates NMDAR abundance	NMDAR formation in the glutamatergic system	[42]
*STAG1*	Stromal antigen 1	Encodes a subunit of the cohesin protein, which is essential for sister chromatid cohesion during mitosis and meiosis	Neuronal maturation and chromatic modification	[46]
*TRIO*	Trio rho guanine nucleotide exchange factor	TRIO is a guanine nucleotide exchange factor (GEF) for RhoA and RAC1 GTPases	Supports excitatory synaptic structure and dysfunctions in CA1 pyramidal cells	[42,48]
*XPO7*	Exportin 7	Role unclear	Not known	[42]
*ZMYND11*	Zinc finger MYND-type containing 11	Regulates the production of different mRNA isoforms in the brain	Neuronal differentiation	[46]
*ZNF136*	Zinc finger protein 136	Encodes a zinc-finger protein with a Krüppel associated Box (KRAB) domain, which may act as a transcriptional repressor	Not known	[46]

## 7. Massively Parallel Sequencing Studies for Schizophrenia in Multiplex Non-Consanguineous Families

Many genetic loci have been identified segregating with schizophrenia in studies involving two or more affected individuals within nuclear families. In a large family with eleven individuals affected with schizophrenia spectrum disorder, chromosomal haplotype 5p11-5q13 was observed to co-segregate in a dominant inheritance pattern in nine out of the eleven affected individuals [49]. In a follow-up study, *SALPR* was identified as a candidate gene, and analysis of its promoter and coding region revealed multiple polymorphisms [50]. A large linkage study was reported the following year involving 409 European or African American families, each with at least two patients who had schizophrenia or schizoaffective disorders. Nonparametric multipoint linkage analysis suggested evidence of linkage at two regions on chromosomes 8p23.3-q12 and 11p11.2-q22.3. Additional analysis revealed two other regions on chromosomes 4p16.1-p15.32 in African American families and at 5p14.3-q11.2 in families with European ancestry [51]. Later, multiple linkage studies were conducted, including a genome scan meta-analysis involving 3255 families with 7413 affected individuals who have schizophrenia or related disorders. This meta-analysis revealed multiple loci linked to schizophrenia, including regions on chromosomes 1, 2q, 3q, 4q, 5q, 8p, and 10q [52]. In a subsequent study, exome sequencing was conducted in five multigenerational multiplex families, which unveiled five protein-altering variants in three distinct genes, *LRP1B*, *GRM5,* and *PPEF2,* that segregated with the disorders in the individual families [53] (Table 3). Of the five pedigrees, three showed distinct missense variants in the same gene, *LRP1B* [53].

In a single Japanese multiplex, multigenerational family, linkage analysis detected two susceptibility loci on chromosomes 3q and 4q [54]. Exome sequencing in the same family identified a rare heterozygous missense variant in *UNC13B* in five of six patients with schizophrenia [55] (Table 3). Follow-up resequencing as a case–control study using 1753 patients and 1602 controls did not reveal any significant association of *UNC13B* variants with schizophrenia in a Japanese population. However, other populations remained unexplored [55]. Exome sequencing in a Chinese family revealed a heterozygous missense variant in *RELN* as a potential highly penetrant risk factor for schizophrenia [56] (Table 3). The significance of *RELN* was further confirmed in another case–control association study from the Chinese Han population consisting of 102 unrelated schizophrenia patients and 169 healthy controls. Significant positive association of four SNPs in *RELN* with schizophrenia was identified in this population [57].

In 2016, nine multigenerational families, each comprising three individuals diagnosed with schizophrenia spectrum disorders, were identified. Comprehensive whole-genome sequencing detected two rare, highly penetrant, segregating missense variants in *SHANK2* and *SMARCA1* in the patients from two families [58] (Table 3). The other seven families remained without a genetic diagnosis [58]. In another family with five individuals exhibiting the condition, a heterozygous frameshift variant in *GRIN3B* was identified as a plausible etiological factor for schizophrenia [59] (Table 3). A heterozygous missense variant in *TENM4* co-segregated with the phenotype in all four affected individuals from a Han Chinese family [60] (Table 3). Subsequent investigation in 120 sporadic schizophrenia patients revealed two additional variants in *TENM4* associated with schizophrenia [60]. Later, homozygous recessive variants in six distinct genes were identified as co-segregating with the symptoms of schizophrenia in two different non-consanguineous families from China. These genes were mostly involved in chromatin remodeling and neuronal development [61] (Table 3).

**Table 3:** Summary of genes implicated in schizophrenia pathogenesis through massively parallel sequencing in non-consanguineous families.

Gene Symbol	Gene Name	Putative Role in Schizophrenia Pathogenesis	Mechanism Involved	Inheritance Pattern	References
*GRIN3B*	Glutamate ionotropic receptor NMDAR subunit	Encodes a protein that forms a heterotetramer with GRIN1 to form the NMDAR	Component of the glutamatergic system	Monoallelic	[59]
*GRM5*	Glutamate receptor subtype 5	Encodes a G protein-coupled receptor that is activated by glutamate and regulates various downstream signaling pathways	Component of the glutamatergic system	Monoallelic	[53]
*LRP1B*	Lipoprotein receptor-related protein 1B	Protein binds with the scaffolding protein PSD-95 and interacts with NMDAR	Glutamatergic system dysregulation	Monoallelic	[53]
*PKHD1L1*	Polycystic kidney and hepatic disease 1-like 1	Encodes a large transmembrane receptor protein named fibrocystin-L, which regulates the expression of KCC2 (potassium-chloride cotransporter)	Neurotransmission	Biallelic	[61]
*PPEF2*	Protein phosphatase with EF-hand domain 2	Encodes a threonine/serine phosphatase with two EF-hand calcium-binding domains and acts as a calcium-sensing regulator	Synaptic transmission	Monoallelic	[53]
*PRKDC*	Protein kinase, DNA-activated, catalytic subunit	Encodes a protein that works with the Ku70/80 heterodimer protein and plays a role in DNA double-strand break repair and recombination	Chromatin remodeling	Biallelic	[61]
*RELN*	Reelin	Encodes a serine protease that plays a role in the layering of neurons	Neuronal migration and neurotransmission	Monoallelic	[56]
*SHANK2*	SH3 and multiple ankyrin repeat domain 2	Encodes a scaffolding protein which interacts with glutamate receptors through its PDZ domain and SH3 domain, and regulates post-synaptic densities of neurons	Glutamatergic system dysregulation	Monoallelic	[58]
*SMARCA1*	SWI/SNF, matric-associated, actin-dependent regulator of chromatin	Encodes an ATPase protein that plays a vital role in chromatin remodeling during transcription	Chromatin remodeling	X-linked	[58]
*SNAI2*	Snail family transcriptional repressor 2	A transcription factor that binds to DNA to repress the expression of other genes. Essential for neural crest cell development, giving rise to nerve tissue	Neuronal development and chromatin remodeling	Biallelic	[61]
*ST18*	Suppression of tumorigenicity 18	A transcription factor that enables sequence-specific DNA binding activity to the RNA polymerase II cis-regulatory region	Neuronal development and chromatin remodeling	Biallelic	[61]
*TENM4*	Teneurin transmembrane 4	Encodes a transmembrane protein that functions as a cell adhesion molecule	Neuronal development and differentiation	Monoallelic	[60]
*TFAP2B*	Transcription factor AP-2 beta	A transcription factor that recognizes specific DNA sequences (consensus 5′-GCCNNNGGC-3′) and either activates or suppresses the expression of target genes. It plays an essential role in neural crest cell migration	Neuronal development and chromatin remodeling	Biallelic	[61]
*TFEB*	Transcription factor EB	A transcription factor that recognizes specific promoter regions (E-boxes) to act as a transcription activator. It plays a role in lysosome formation and autophagy.	Chromatin remodeling	Biallelic	[61]
*UNC13B*	Unc-13 homolog B	Encodes a protein that plays a crucial role in synaptic vesicle exocytosis, specifically in the priming and fusion of vesicles at presynaptic terminals	Neurotransmission	Monoallelic	[55,62]

## 8. Variants Reported for Schizophrenia in Multiplex Consanguineous Families

Gene variants with individually large effects in causing schizophrenia are uncommon, but they can be identified in some multiplex families, as evident from the studies described above. Consanguineous populations are more likely to yield such genes/loci in recessive cases [63,64,65]. However, many dominantly inherited genes/loci have been identified in families expected to have a preponderance of recessive alleles. For instance, exome sequencing of three consanguineous Iranian families with twelve individuals affected by schizophrenia revealed rare missense, heterozygous, protein-altering variants in *ANKK1* and *ANK3* [66] (Table 4). A study of a Pakistani consanguineous family with multiple affected members revealed rare, heterozygous variants with high penetrance. Two novel missense variants were found in *GRIN2A* and *NRG3*, both of which segregated with the symptoms of schizophrenia [67] (Table 4). In another instance, rare compound heterozygous variants in *SPATA7* were identified as segregating in two affected siblings through exome sequencing [68] (Table 4).

Several instances exist where rare, damaging recessively inherited variants with complete penetrance were found associated with schizophrenia in consanguineous families. A locus on chromosome 22q12.3-q13.3 was mapped for recessively inherited schizophrenia in a Pakistani consanguineous family with recessively inherited schizophrenia; however, the specific gene or variant was not identified [69]. Later, a few homozygous or hemizygous, recessive, missense variants in five genes were associated with schizophrenia in consanguineous Pakistani families [70,71,72] (Table 4).

**Table 4:** Summary of genes with rare variants associated with schizophrenia segregating in multiplex consanguineous families.

Gene Symbol	Gene Name	Putative Role in Schizophrenia Pathogenesis	Mechanism Involved	Inheritance Pattern	References
*ANK3*	Ankyrin 3	With its spectrin-binding domain, ANK3 links integral membrane proteins with the underlying spectrin–actin cytoskeleton	Neuronal differentiation and development	Monoallelic	[66]
*ANKK1*	Ankyrin repeat kinase containing	Encodes a protein belonging to the Ser/Thr kinase family, which is involved in signal transduction pathways through interacting with DRD2	Dopamine and glutamate interactions regulate NMDAR expression	Monoallelic	[66,73]
*GRIN2A*	Glutamate receptor ionotropic N-methyl-D-aspartate subunit 2A	Encodes a NMDAR that increases calcium influx in neurons and regulates neuronal signaling	Component of the glutamatergic system	Monoallelic	[67]
*IL1RAPL1*	Interleukin 1 receptor accessory protein-like 1	Encodes a protein that binds with PSD-95 and Mcf2l and connects the pre-synapse with the post-synapse	Glutamatergic system dysregulation	X-linked	[70]
*INSR*	Insulin receptor	Encodes a protein that binds to insulin circulating in the bloodstream	Neurotransmission	Biallelic	[72]
*NFXL1*	Nuclear transcription factor, X-box binding-like 1.	Encodes a transcription factor that binds DNA and regulates gene expression for RNA polymerase II-specific genes	Predicted to regulate chromatin remodeling at the centromere	Biallelic	[72]
*NRG3*	Neuregulin 3	Encodes a protein that interacts with the ErbB4 receptor and guides the migration and placement of interneurons	Regulated glutamate release by interacting with the SNARE complex	Monoallelic	[67,74]
*RGS3*	Regulator of G protein signaling 3	Increase the GTPase activity of the alpha subunits of G proteins, promoting their conversion from the active GTP-bound form to the inactive GDP-bound form	Regulates glutamatergic synaptic transmission across sensory neurons	Biallelic	[70,75]
*SPATA7*	Spermatogenesis-associated protein 7	Role unclear	Not known	Biallelic	[68]
*USP53*	Ubiquitin-specific peptidase 53	A member of the ubiquitin-specific peptidase (USP) family, in which enzymes are crucial for maintaining ubiquitination and deubiquitination	Co-localized with glutamatergic system components GRIP2 and GluA2, but the mechanism is not known	Biallelic	[71]

## 9. De Novo Variants in Schizophrenia

De novo mutations in various genes have been implicated in schizophrenia. Exome sequencing of 53 sporadic cases in parent–proband trios identified 40 genes with rare, protein-altering de novo variants in 27 cases. However, among these 40 genes, only those within *DGCR2* were predicted to be the most pathogenic [76] (Table 5). Subsequent studies have identified multiple genes with de novo mutations, including both missense and loss-of-function variants [77,78,79] (Table 5).

**Table 5:** Genes with de novo mutations, reported/predicted as the most disruptive and implicated in schizophrenia pathogenesis through trio studies.

Gene Symbol	Gene Name	Putative Role in Schizophrenia Pathogenesis	Mechanism Involved	References
*CHD2*	Chromodomain DNA helicase binding protein 2	Regulates gene expression by influencing the structure of chromatin	Chromatin remodeling	[79]
*DGCR2*	DiGeorge syndrome critical region gene 2	Putative adhesion receptor possibly involved in cell–cell or cell–matrix interactions required for normal cell differentiation and migration	Neuronal development	[76]
*DPYD*	Dihydropyrimidine dehydrogenase	Involved in the metabolism of uracil and thymine	Chromatin remodeling	[77]
*LAMA2*	Laminin subunit alpha-2	Encodes laminin, which mediates the attachment, migration, and organization of cells into tissues during embryonic development	Synaptic plasticity	[77]
*SETD1A*	SET domain containing 1A, histone lysine methyltransferase.	Encodes a protein which acts as a histone methyltransferase, adding methyl groups to lysine 4 of histone H3 (H3K4), which is associated with gene activation	Chromatin remodeling	[78]
*TRRAP*	Transformation/transcription domain-associated protein	A component of histone acetyltransferase (HAT) complexes	Chromatin remodeling	[77]
*VPS39*	Vacuolar protein sorting 39	Essential for the fusion of vesicles and organelles, particularly in the endolysosomal pathway	Neuronal development	[77]

## 10. Microdeletions and Copy-Number Variants in Schizophrenia

Several microdeletions are associated with schizophrenia. In an analysis comprising two individuals, a mother and daughter, affected with schizophrenia, a heterozygous microdeletion was detected on chromosome 14q13, which encompassed the third intron of *NPAS3* [80] (Table 6). Haploinsufficiency was hypothesized to cause the phenotype in both. Interestingly, the daughter exhibited more pronounced symptoms and also had a second microdeletion of a non-coding element on chromosome 9q34 [80]. In another family, a heterozygous microduplication involving *HOMER*, *RasGRF2*, and *CMYA5* was found segregating with the symptoms of schizophrenia [67]. A loss-of-function of *OTUD7A* deletion on chromosome 15q13.3 was also implicated in schizophrenia [45]. In a multigenerational family with multiple patients, two rare deletions on chromosome 2p16.2 were found to segregate with the disorder. The 2p16.2 locus has been reported to play a significant role in DNA regulation, transcription factor binding, chromatin remodeling, and regulation of the non-coding mRNA *AK127244* [81].

Copy number variants have also been significantly associated with the genetic etiology of schizophrenia. In a case–control study involving Ashkenazi Jews, a copy-number variant on chromosome 3q29 was associated with schizophrenia in 245 patients versus 490 controls, which was further confirmed in a larger cohort of 7545 schizophrenic subjects and 39748 controls [82] (Table 6). A comprehensive GWAS involving 21094 individuals who have schizophrenia compared to 20227 controls revealed global enrichment of copy-number variants in patients versus controls [83] (Table 6). A gene set burden test revealed that genes affected by these copy-number variants were associated with synaptic functions and distinct neurobehavioral phenotypes in mouse models [83]. Rare copy number variations at chromosomes 15q11.2-q13.1 and 16p11.2 have also been linked to treatment-resistant psychosis [39], whereas those on chromosome 16p11.2 were associated with schizophrenia in 4551 patients versus 6391 controls [84].

**Table 6:** Copy-number variants associated with schizophrenia through case–control or familial studies.

Copy-Number Variant (CNV)	Chromosomal Change	Impacted Gene	Putative Role of Gene in Schizophrenia Pathogenesis	References
1q21.1	Del	NA	NA	[83,85]
1q21.1	Dup	NA	NA	[83,85]
2p16.3	Del	*NRXN1*	Synapse formation	[83,85]
3q29	Del	NA	NA	[82,83,85]
7q11.21	Del	*ZNF92*	DNA-binding transcription factor	[83]
7q11.21	Dup	*ZNF92*	DNA-binding transcription factor	[83]
7q11.23	Dup	NA	NA	[83,85]
7p36.3	Del and Dup	*VIPR2*; *WDR60*	VIPR2 regulates neurogenesis; WDR60 regulates neuronal migration and microtubule organization	[83]
8q22.2	Del	*VPS13B*	Sorts and transports proteins in the nervous system	[83]
9p24.3	Del and Dup	*DMRT1*	DNA-binding transcription factor	[83]
13q12.11	Dup	*ZMYM5*	Transcriptional regulator	[83]
15q11.2	Del	NA	NA	[83,85]
15q11-q13	Dup	NA	NA	[85]
15q11.2-q13.1	Dup	NA	NA	[39]
15q13.3	Del	NA	NA	[83,85]
16p11.2 (distal)	Del	NA	NA	[83]
16p11.2	Dup	NA	NA	[39,83,85]
16p12.1	Del	NA	NA	[85]
16p13.11	Dup	NA	NA	[85]
22q11.2	Del	NA	NA	[39,83,85]
22q11.21	Dup	NA	NA	[83]
Xq28 (distal)	Dup	NA	NA	[83]
Xq28	Dup	*MAGEA11*	Encodes a protein that acts as an androgen receptor coregulator	[83]

Del—deletion; Dup—duplication; NA—not available.

## 11. Exploring the Potential Roles of Candidate Genes in Model Systems

Investigations of candidate genes identified using GWAS, case studies, large cohorts, and trio sets, as well as the exploration of genes with large-effect variants in multiplex families, implicate common molecular mechanisms that may play a significant role in the pathophysiology of schizophrenia. In some cases, pertinent in vitro and in vivo models have been studied, utilizing either heterozygous or homozygous knockouts. By exploring these animal or cell line model systems, additional deductions regarding the molecular pathways have been determined.

### 11.1. Glutamatergic System Dysregulation

**Figure 1:**
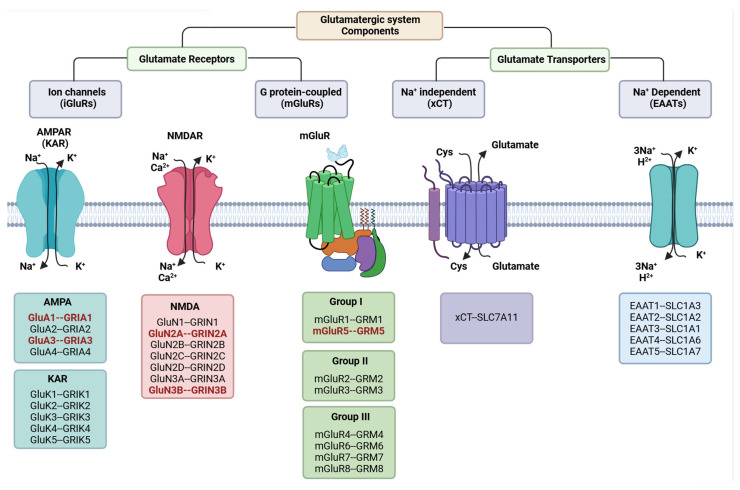
Glutamatergic system components. The left side shows subunits of ion channels (iGluRs), including alpha-amino-3-hydroxy-5-methyl-4-isoxazolepropionic acid receptors (AMPARs), and N-methyl-D-aspartate receptors (NMDARs), and their coding proteins. The middle panel shows the proteins, grouped into three categories associated with metabotropic glutamate receptors (mGluRs). The right panel indicates Na+-dependent and Na+-independent glutamate transporters and proteins [86]. Multiple components of the glutamatergic system can be dysregulated in schizophrenia, particularly those components related to AMPAR, NMDAR, and mGluR. The acronyms highlighted in red indicate proteins that are components of the glutamatergic system and are dysregulated in schizophrenia.

**Figure 2:**
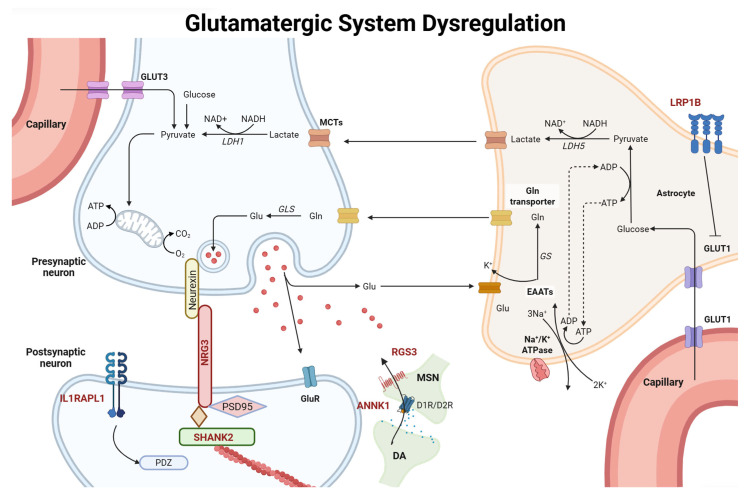
Schematic representation of molecular and cellular components of the glutamatergic system susceptible to dysregulation in schizophrenia. The glutamatergic system involves the uptake, transport, metabolism, and regulation of glutamate in neurons and astrocytes [90]. Presynaptic neurons synthesize glutamate (Glu) from glutamine (Gln) via glutaminase (GLS) and release Glu to the synaptic cleft. Postsynaptic neurons receive Glu via glutamate receptors (GluR). Capillaries supply glucose via glucose transporter 1 (GLUT1) to astrocytes and via glucose transporter 3 (GLUT3) to neurons [90]. Multiple scaffolding and anchoring proteins are involved in this process, including PSD95, SHANK2, NRG3, and IL1RAPL1 [91,92]. ANNK1 interacts with the glutamatergic system via dopamine signaling [93], whereas RGS3 regulates G protein-coupled receptor signaling by modulating N-type calcium channels [75]. LRP1B inhibits GLUT1 in astrocytes [94]. *SP4* and *SRR* are also implicated in disrupting the glutamatergic pathway [95,96], whereas *TRIO* is also implicated in disrupting the glutamatergic pathway [95,96] via an unknown mechanism. Acronyms in red indicate proteins that are directly or indirectly implicated in the glutamatergic system. GluR, glutamate receptor; GS, Glutamate synthetase; EAATs, excitatory amino acid transporters; GluA, AMPA receptor subunit A; LDHS, lactate dehydrogenase; MCTs; monocarboxylate transporters; DA; dopaminergic neuron; MSN; medium spiny neuron; D1R, D1 receptor; D2R, D2 receptor.

We found compelling evidence for the involvement of the glutamatergic pathway as the underlying mechanism for the majority of cases of schizophrenia. Protein products of some of the genes are essential components of this pathway, including *GluA1*, *GluA3*, *GluN2A*, *GluN3B,* and *mGluR5* (Figure 1), whereas others, such as *IL1RAPL1*, *NRG3*, and *SHANK2,* were directly linked to this pathway (Figure 2). For most of these genes, knock-out animal model studies have shown significant effects on animal behavior. Knockout of other genes, such as *ANNK1*, *LPR1B*, *RGS3*, and *SP4,* indirectly affects the pathway.

Glutamate is the primary excitatory neurotransmitter in the central nervous system. Glutamate, along with its target, the NMDAR, plays a central role in essential brain functions, including neural plasticity, the development of neural networks, and learning and memory. Conversely, high glutamate concentrations are associated with excitotoxicity and neuronal degeneration [87]. The glutamatergic system has been implicated in the pathophysiology of schizophrenia, particularly involving the NMDAR [87]. The “glutamate hypothesis of schizophrenia” suggests that hypofunction of NMDAR contributes to the positive (hallucinations, delusions), negative (social withdrawal, lack of motivation), as well as cognitive deficits associated with the disorder [88,89].

*ANNK1*, *GRIA1*, *GRIA3*, *GRIN2A*, *GRIN3B*, *GRM5*, *IL1RAPL1*, *LRP1B*, *NRG3*, *RGS3*, *SHANK2*, *SP4*, *SRR*, and *TRIO* encode protein products that are implicated directly or indirectly in NMDAR formation or function. Some of these are direct components of the glutamatergic system (Figure 1), whereas others can modulate it indirectly (Figure 2). Prior research also implicates NMDAR disruption with schizophrenia. The acute administration of NMDAR antagonists such as phencyclidine (PCP), ketamine, or methamphetamine (METH) has the capacity to elicit schizophrenia-like symptoms in healthy human subjects, as well as regressive symptoms in patients who have schizophrenia [97].

Experiments across different mouse models for genes implicated in this pathway have yielded vital insights. For instance, studies in the mouse model Ankk1Δ-D2RN showed that glutamatergic transmission was enhanced in D2R-SPN (D2 receptor-expressing spiny projection neurons) due to downregulation of Ankk1, which increased neuronal excitability [98]. In another study, *Gria2* knockout mice had impaired stimulus-reward learning, which is necessary for Pavlovian-to-instrumental transfer. The presence of GRIA2 inhibits calcium influx via alpha-amino-3-hydroxy-5-methyl-4- isoxazolepropionic acid receptors (AMPA receptor; AMPAR) through glutamate signaling, whereas the deletion of this subunit enhances hippocampal long-term potentiation [99]. 

The *GRIA2* paralog gene, *GRIA3,* encodes the GluA3 subunit of AMPAR, and the impairment of AMPAR function has been linked to the pathophysiology of schizophrenia. Gene knockout models targeting exons 1 and 2 and intron 1 of Gria3 in mice have been established, suggesting a compromise in excitatory neurotransmission specifically within the medial prefrontal cortex, accompanied by diminished neuronal activity [100]. These knockout mouse models exhibited aggressive behavior. Excitingly, these can be alleviated by introducing an adeno-associated viral vector that expresses *GluA3* in the medial prefrontal cortex [100].

Another implicated gene, *GRIN2A*, encodes a glutamate-activated ion channel, a component of the NMDAR subunit. In a mouse model in which the C-terminal domain of *Grin2a* was selectively deleted (*Grin2a*^ΔC/ΔC^), the deletion affected NMDAR channel function and impacted the glutamatergic pathway. Mutant mice showed dysregulated attention, a deficit in hippocampus-dependent short-term memory, and impairment of hippocampal synaptic plasticity. However, the mice were capable of forming long-term memories [101]. In contrast, N-terminal knock-in mice carrying *Grin2a*N615S exhibit reduced hippocampal activity, audiogenic seizures, attention deficit, and impaired associative learning [102].

GPRIN3 mediates dopamine receptor D2R in subcortical basal ganglia. *Gprin3*/*Grin3* knockout mice show symptoms of increased anxiety-like behavior and reduced locomotor activity in the elevated maze test [103], although another study assessing anxiety-like behavior using a light/dark apparatus did not detect that effect [104]. GRM5/mGluR5 is a G protein-coupled receptor for the excitatory neurotransmitter L-glutamate. *Grm5* knockout mice (whether homozygous or heterozygous for null mutations) exhibit disturbed locomotor and digging behavior, are hyperactive, and display anxiety-like behaviors [105]. A player in the synaptic function is IL1RAPL1, which interacts with postsynaptic density-95 (PSD-95) and maintains glutamatergic synapses by regulating the trafficking of AMPA receptors [106]. IL1RAPL1 knockout mouse models have reduced spine densities and manifest deficits in cognitive acquisition, behavioral adaptability, motor activity, and regulation of anxiety [106]. NRG3, a neuregulin, interacts with the SNARE complex and regulates L-glutamate release to the synapse, ultimately controlling synaptic function [74]. *Nrg3*^−/−^ knockout mouse model also displays different neurobehavioral phenotypes resembling the symptoms of psychosis, including deficient fear conditioning, impaired ability to suppress the acoustic startle response, and novelty-induced hyperactivity [107]. *RGS3* encodes a negative regulator of G protein signaling and has so far been implicated with schizophrenia in just one family. The *rgs3* mutant in *C. elegans* does not respond to intense sensory stimuli. They have aberrantly increased G protein-coupled calcium signaling and reduced synaptic output, leading to behavioral deficits. Interestingly, these can be restored by enhancing glutamatergic synaptic transmission across sensory neurons [75].

SHANK2 functions as a scaffolding protein within the post-synaptic density, facilitating the anchoring of a protein complex that interlinks NMDAR, AMPAR, and mGluR [108]. In different studies, multiple knockout mouse models, including *Shank2*Δex16 (with exon 16 deletion) [109] and *Shank2*Δex15-16 (with exon 15–16 deletion) [110], manifested cognitive and social impairments [111]. SP4 encodes a transcription factor, and the *Sp4* hypomorphic mouse serves as a valuable model for understanding the proposed NMDAR-specific hypoglutamatergic impairment, considered fundamental to the etiology of schizophrenia [97]. Expression of *Sp4* in mice is significantly elevated in the hippocampal CA1 region. Compared with wild-type mice, *Sp4* hypomorphic mutants showed impaired long-term potentiation in the hippocampal CA1 region and reduced spatial learning and memory [97].

Serine binds to NMDAR, and *SRR* encodes a serine racemase which synthesizes D-serine. *Srr* knock-out mice have reduced NMDAR activity and exhibit many neurochemical and behavioral deficits associated with schizophrenia, including reduced inhibitory synapses in CA1 pyramidal neurons, enhanced synaptically driven neuronal excitability, and loss of GABAergic synapses in pyramidal neurons [95]. *TRIO* is another gene that plays a pivotal role in glutamatergic neurotransmission and long-term potentiation, in conjunction with its paralog, Kalirin [96]. Long-term potentiation enhances the efficacy of glutamatergic synapses, thereby affecting synaptic plasticity, a fundamental mechanism hypothesized to underlie memory and learning processes [48]. TRIO loss-of-function mutants have disturbed dendritic arborization, axonal guidance, and synaptic neurotransmission [112].

### 11.2. Dysregulation of Chromatin Modification

Chromatin modification, also known as epigenetic regulation, involves altering gene expression without changing the DNA sequence. This process can turn a gene “on” or “off”, ultimately regulating cellular processes. Epigenetic regulation potentially plays a significant role in the pathophysiology of schizophrenia [113]. Multiple studies have reported dysregulated components of histone modification pathways in patients affected with schizophrenia. These include increased levels of histone 3-(methyl) arginine 17 in the prefrontal cortex and hippocampus of schizophrenia patients; abnormal expression of histone deacetylase 3 (HDAC3) in the temporal cortex in postmortem brains of schizophrenia patients; and dysregulation of many histone-related genes in the blood of schizophrenia patients [114].

The genes identified through genetic studies as impacting chromatin remodeling in schizophrenia are *CHD2*, *DPYD*, *NFXL1*, *PRKDC*, *SETD1A*, *SMARCA1*, *SNAI2*, *ST18*, *TFAP2B*, *TFEB,* and *TRRAP*. Mice models for some of these genes have revealed behavioral and neurological effects. For example, mice with heterozygous *Chd2* variants have impaired neuronal proliferation and altered neuronal excitability, including changes in excitatory and inhibitory synaptic function. In vivo experiments revealed that *Chd2*^+/−^ mice display abnormal cortical rhythmogenesis, neurogenesis, synaptic transmission, and deficits in long-term memory, aligning with human psychosis phenotypes [115]. *Dpyd* knockout mice have markedly reduced sleep compared to the wild-type littermate controls, whereas no difference in other measured behaviors was noted [116]. *NFXL1* encodes a protein that acts as a transcriptional activator and regulates the gene expression of other genes. *Nfxl1* knock-out mice exhibit decreased body weight and body length, along with skeletal abnormalities (https://www.informatics.jax.org/; accessed on 1 September 2025). However, behavioral defects remain unexplored. *PRKDC* interacts with *GTF21,* which is known to regulate DNA repair pathways. *PRKDC*, *GTF21,* and *BRCA1* are present on chromosome 7q11.23, a region that is known to play a significant role in early and late fetal development [117].

The haploinsufficiency murine model of *Setd1a* (*Setd1a*^+/−^) has been widely studied to elucidate the epigenetic mechanisms proposed to contribute to schizophrenia pathophysiology. Studies revealed that *Setd1a*^+/−^ mice exhibit an approximate 50% reduction in the encoded protein levels in frontal brain regions. Further investigations into the medial prefrontal cortex and striatum of these mice revealed morphological defects in dendrites, abnormal synaptic development, and exocytosis impairment. Moreover, these *Setd1a*^+/−^ mice displayed schizophrenia-like behavioral phenotypes, including impaired social interactions, sensorimotor gating deficits, and working memory impairments [118].

*Smarca1*^Ex6DEL^ mutant mice (with exon 6 deletion) displayed forebrain hypercellularity and enlarged brain, enhanced progenitor cell expansion, and delayed differentiation via chromatin modification [119]. *SNAI2* is a gene that encodes the Snail family transcriptional repressor 2 (C2H2), acting as a regulator of epithelial-to-mesenchymal transition (EMT) [120]. Mediation of a knockdown with short hairpin RNA (shRNA) in a human cell line and of a deletion with CRISPR/Cas9 in a murine N1E-115 cell line revealed that SNAI2 promotes cellular differentiation [120]. These genetic changes recruited chromatin remodelers and DNA methyltransferases to target gene promoters, thereby regulating EMT and promoting cellular differentiation [121]. Another implicated gene in schizophrenia is *ST18* [61]. Mutant mice with loss-of-function in *St18* (−/−) showed fewer projection neurons in the globus pallidus pars externa (GPe). Transcription factor *St18* null embryos indicate that ST18 guides medial ganglionic eminence (MGE) progenitor cells towards developing a large expansion of nascent cortical neurons, while reducing the formation of GPe neurons [122]. Recently, transcriptomic analysis also confirmed its function [123]. However, no behavioral studies in animal models have been described. ST18 is not directly involved in chromatin modification. However, its expression is mediated by H3K9me2 (histone H3 lysine 9 dimethylation), a crucial epigenetic mark regulated by EHMT1 (euchromatic histone methyltransferase 1) for epigenetic regulation [124].

The transcription factor TFAP2 is associated with schizophrenia and is a pioneer factor (a transcription factor with access to chromatin) that has been shown to drive epigenomic remodeling to regulate neural crest specification during embryonic development [125]. Heterozygous mice *Tfap2a*^(+/−)^ exhibit mild developmental defects in craniofacial and brain development [126], whereas another *Tfap2b*^(+/−)^ mutant allele causes developmental defects [127]. Recently, these mutant mice exhibited disturbed circadian rhythms and impaired stress resistance, leading to memory loss [128]. Another gene, *TRRAP*, encodes a scaffold protein that plays a crucial role in recruiting several multiprotein histone acetyltransferase (HAT) complexes for DNA binding and chromatin modification [129]. *Trrap* deletion in Purkinje neurons in a mouse model (referred to as *Trrap*-PCΔ) causes an age-dependent loss of existing neurons, axonal swellings, dendritic retraction, locomotor dysfunction, ataxia, impaired coordination, and an unsteady gait, along with multiple other neuronal defects, leading to neurodegeneration [130].

In summary, although multiple genes are associated with schizophrenia, experimental analysis was only performed for *CHD2*, *SETD1A*, *SMARCA1*, *SNAI2*, *TFAP2B*, *TFEB,* and *TRRAP* to implicate their direct role as chromatin modifiers in the nervous system. Different mutations in these genes impacted the behavior in animal models. However, other genes known to participate in chromatin modification, such as *DPYD*, are associated with schizophrenia in humans; no animal models of this gene with behavioral deficits are known to date.

### 11.3. Ubiquitin–Proteasome System Impairment

The ubiquitin–proteasome system is integral to maintaining neuronal homeostasis by degrading proteins and is known to regulate synaptic development, maintenance, and plasticity [131]. The impairment of this degradation system has been correlated with a range of neurological disorders, including schizophrenia, autism, and other neurodevelopmental disorders [131]. Components of the ubiquitin–proteasome system, especially E3 ligases, are important because they determine the ubiquitination targets for degradation in various diseases [132]. The genes *AKAP11*, *CUL1*, *HERC1*, and *OTUD7A* associated with schizophrenia have key roles in the ubiquitin–proteasome system. For example, *AKAP11* is a schizophrenia risk gene and is also known as a modulator of neuronal synaptic plasticity [133]. The phosphodegron motif (degron Thr-Pro-Pro-Xaa-Ser sequence with phosphorylated Thr and Ser residues) present on the surface of AKAP11 is recognized by box protein W7 (FBXW7), which targets it for degradation by the ubiquitin–proteasome system [133]. *Akap11* knockout mice (exon 2–6 deletion) had symptoms of anxiety, compromised sensorimotor gating, and schizophrenia-related synaptic dysfunction [134].

CUL1 is also a component of the SCF complex (a component of Skp1-Cullin-F-box) of E3 ubiquitin ligases. *Cul1* knockout murine models exhibit reduced expression of SCF ubiquitin ligases, but no study has examined their effects on behavioral phenotypes or other brain-related changes [132]. Another gene part of this system is *HERC1*. *Herc1* knockout mice showed cerebellum-related spatial learning alterations, impairments in hippocampal learning and memory, and alterations in the size and density of dendritic spines on neurons of the lateral amygdala [131]. An important gene encoding a deubiquitinating enzyme (DUB) is *OTUD7A*, a major regulatory gene in the 15q13.3 microdeletion syndrome, which includes symptoms such as intellectual disability, autism spectrum disorder, epilepsy, and schizophrenia [135]. *Otud7a*-null mice exhibit several characteristics, including decreased body weight, delayed development, irregular electroencephalography patterns, seizures, reduced ultrasonic vocalizations, diminished grip strength, impaired motor learning and coordination, and decreased acoustic startle [136]. The *Otud7a*-null mice exhibit reduced dendritic spine density compared to wild-type littermates. Additionally, the frequency of miniature excitatory postsynaptic currents (mEPSCs) was reduced in the frontal cortex of *Otud7a*-null mice, consistent with OTUD7A regulating dendritic spine density [136].

In summary, multiple studies identified genes implicated in the ubiquitin-proteosome system. Some of them were also curated using animal models such as *Akap11*, *Herc1*, and *Otud7a*, knock-out mouse models. These models highlighted multiple sensory or motor impairments. However, *Cul1* has not been explored functionally regarding its role in schizophrenia or behavioral phenotypes.

## 12. Limitations and Challenges

Although common variants detected by GWAS explain only a limited proportion of schizophrenia heritability, they may reflect the genetic variability observed among individuals or populations. Secondly, GWAS exhibit bias, as the data largely comprise patients of European ancestry. Thus, the outcomes reflect reduced generalizability of GWAS findings. Substantial inclusion of diverse ethnic groups will enhance global understanding of genetic risk factors. Thirdly, the common alleles identified by GWAS individually confer relatively small schizophrenia risk, with an odds ratio less than 1.2, but collectively, they are estimated to explain around 25% to 50% of the variance in genetic susceptibility [36]. In other words, a large proportion of variance in genetic liability is unaccounted for by the “common disease-common variant” hypothesis [38]. On the other hand, variants predicted as damaging under the “common disease-rare variant” hypothesis do not imply pathogenicity or a causal disease phenotype. We cannot rely only on computational prediction scores without functional studies. Furthermore, rare variants also do not directly point towards the mechanism of disease. To solve this issue, recent American College of Medical Genetics (ACMG) criteria recommend using combined computational, functional, demographic, and clinical data for variant annotation. This recommendation again highlights the importance of functional work for variant assessment [137].

Of note, environmental factors play a significant role in developing schizophrenia and typically remain unaddressed in genetic studies. According to the neural diathesis-stress model of schizophrenia, there is a preexisting vulnerability present for psychosis development starting from prenatal through late childhood [138]. Multiple behavioral markers indicate this vulnerability, including lower general cognitive power, minor physical anomalies, childhood trauma, developmental delays, and parental instabilities [138]. These models argue that adolescence marks critical periods of myelination, hormonal development, gray matter pruning, and neural plasticity [139]. Thus, the role of the environment should be incorporated into genetic studies for a fuller explanation of the development of schizophrenia.

## 13. Translational Implications

Schizophrenia is a complex, multifactorial, neuropsychiatric disorder with many etiological factors, including genetic vulnerability, environmental stresses, and neurodevelopmental alterations [140]. Despite decades of research, how these factors contribute to developing schizophrenia and stratifying subtypes remains only partially understood. Individuals’ polygenic risk scores (PRS) from GWAS are measures of the net influence of thousands of common, small-effect alleles on disease risk. In schizophrenia, higher PRS have significantly predicted higher symptom scores in three out of four independent cohorts of patients with first-episode psychosis [141]. Patients with higher PRS tended to have less improvement with antipsychotic drug treatment compared to patients with low PRS. These results suggest the potential use of PRS as a prognostic biomarker [141]. However, another study did not support PRS use for the determination of risk assessment in patients with treatment-resistant schizophrenia [142].

Several factors could improve PRS estimation in the future. For example, GWAS with more patients and controls could yield greater effect sizes, supporting the identification of common alleles and providing a better understanding of their role in risk assessment. Biomarkers are also needed. Neuroimaging data have predicted high-risk individuals who have a greater tendency to develop psychosis or schizophrenia [143]. Similarly, electrophysiological changes measured using electroencephalography (EEG) have the potential to serve as biomarkers of the full spatiotemporal dynamics of neural activation associated with a wide variety of cognitive processes, including psychosis/schizophrenia [144]. PRS estimation, when combined with other environmental factors, neuroimaging, and electrophysiological measurements, may enable early diagnosis of schizophrenia, patient stratification, and personalized medicine [38].

The biological heterogeneity inherent in the clinical diagnosis of schizophrenia also impairs both preventative and individualized care. Patient stratification on the basis of clinical presentations such as paranoid, disorganized, and catatonic subtypes could also help differentiate among distinct subtypes for targeted therapies. However, these symptom subtypes were removed from DSM-5 due to limited diagnostic stability, low reliability, and poor validity [145]. Along with symptom subtype, symptom severity, and duration, it could more effectively support patient classification. For example, a recent clinical assessment using smartphone-based digital phenotyping identified three well-defined clusters in a small cohort of 74 patients [146]. In the future, similar pipelines for larger datasets could be devised to transparently address missing-data challenges and scale up across subtypes to uncover schizophrenia heterogeneity.

Interestingly, the three major pathways (glutamatergic system dysregulation, chromatin modification, and ubiquitination) emerging from genetic studies of schizophrenia interact with each other. For example, *ANKK1* is expressed in D2 medium spiny neurons, and variants in this gene affect the dopaminergic system, but they also indirectly modulate NMDARs in the glutamatergic system [98]. Similarly, ubiquitination of the GluA1 subunit of AMPAR in the glutamatergic system directly impacts memory, synaptic plasticity, and cognitive flexibility in knock-in mouse models [147]. These results imply that these pathways are strongly interconnected, and disturbances in one pathway may ultimately lead to dysregulation across multiple pathways.

## 14. Conclusions

Evidence derived from disparate research indicates the existence of common pathways in most cases of schizophrenia. While the majority of individual genetic variations may exert minimal effects, their cumulative influence significantly affects essential neurobiological processes, particularly those associated with synaptic function, dysregulation of the glutamatergic system, the ubiquitin–proteasome pathway, and neuronal development. Although a multitude of genetic variants and specific genes have been correlated with schizophrenia over the past 20 years, extreme heterogeneity has prevented replicable, reproducible research on the effects of candidate genes [148]. Numerous investigations identified genes that co-segregate with schizophrenia; however, they frequently fail to rigorously explore the functional implications of these genes in the context of the disorder. Additional research is imperative to reconcile the disparity between genetic association findings and a comprehensive biological understanding. Furthermore, as long-read sequencing technology has revolutionized genome science, the technique should also be used to detect structural variants, elucidate complex architectures, and advance understanding of non-coding regions and regulatory elements in the pathophysiology of schizophrenia [149].

Further research integrating genetic findings with neurobiological experiments is crucial for deciphering the specific disease mechanisms of schizophrenia. Future studies to elucidate the functional roles of identified genes and genetic variants in human-derived iPSC neurons or brain organoids will be vital. These studies should include investigating how these genes influence synaptic function, neuronal development, and other key neurobiological processes implicated in schizophrenia. Specifically, we recommend using CRISPR-Cas9 to generate knock-in or knock-out animal models and performing multiple behavioral tests to delineate the underlying role of genetic variants. Integrating genetic data with other types of “omics” data, such as transcriptomics, proteomics, and metabolomics, can also provide a more comprehensive understanding of the molecular mechanisms underlying schizophrenia. Expanding genetic studies to include diverse populations, especially from understudied ethnic groups, will be very helpful. Future genetic studies of schizophrenia from founder populations could also help in identifying gene variants with large effects that are causal in schizophrenia. By targeting the core mechanisms, such as glutamatergic neurotransmission, synaptic function, or ubiquitination, scientists could address the underlying pathophysiology of schizophrenia, rather than aiming only to treat the symptoms. This strategy could potentially benefit a larger number of patients, even if they have different genetic predispositions. Targeting downstream mechanisms may also bypass some of the genetic heterogeneity associated with schizophrenia and pinpoint the core targets for therapeutics.

## Figures and Tables

**Table 1:** Genes implicated by genome-wide association studies are significantly associated with schizophrenia.

Gene Symbol	Gene Name	Putative Role in Schizophrenia Pathogenesis	Mechanism Involved	References
*BCL11B*	B-cell lymphoma/leukemia 11B	A transcription factor that regulates the development and function of diverse neuronal populations	Neuronal differentiation and development	[8]
*CACNA1C*	Voltage-dependent L-type calcium channel subunit alpha-1C	The pore-forming alpha-1C subunit of the voltage-gated calcium channel is responsible for generating L-type calcium currents	Calcium signaling and neuronal activity	[8]
*CACNA1I*	Calcium channel voltage-dependent alpha-1I subunit	The pore-forming alpha-I subunit of the voltage-gated calcium channel is responsible for generating calcium currents	Calcium signaling and neuronal activity	[8]
*CACNB2*	Calcium channel voltage-dependent β-2 subunit	Forms a voltage-sensitive, dihydropyrimidine (DHP) activated calcium channel, which operates in conjunction with the CACNA1D subunit to modulate the function of the channel throughout neuronal networks	Calcium signaling and neuronal activity	[8]
*DAOA*	D-amino acid oxidase activator	Encodes a protein that functions as an activator of D-amino acid oxidase enzyme, which degrades the gliotransmitter D-serine, a potent activator of NMDAR	Glutamatergic system dysregulation	[35]
*DRD2*	Dopamine receptor D2	Encodes transmembrane G protein-linked receptor on dopaminergic neurons and functions to attenuate the secretion of dopamine	Dopamine and glutamate interactions	[36]
*FAM120A*	Family with sequence similarity 120A	Encodes an RNA-binding protein and is an important constituent of the stress-induced signaling cascade; peroxisome proliferator-activated receptor (PPAR) signaling pathway	Neuronal development	[8]
*FOXP1*	Forkhead box protein P1	Transcriptional regulator of B-cell development	Neuronal development	[8]
*GRIA1*	Glutamate ionotropic receptor AMPA type subunit 1	Encodes the GluA1 subunit of the AMPA receptor, which serves as a fundamental element of excitatory synapses	Glutamatergic system dysregulation and synaptic plasticity	[36]
*GRIN2A*	Glutamate receptor ionotropic N-methyl-D-aspartate subunit 2A	Encodes a N-methyl-D-aspartate receptor (NMDAR), which increases calcium influx in neurons and regulates neuronal signaling	Glutamatergic system dysregulation	[36]
*GRM3*	Glutamate receptor metabotropic 3	A G protein-coupled receptor that performs an essential function in regulating synaptic glutamate transmission	Glutamatergic system dysregulation and synaptic plasticity	[36]
*MYT1L*	Myelin Transcription Factor 1	Transcription factor regulating expression of non-neuronal genes	Neuronal differentiation and maturation	[8]
*RERE*	Arginine-glutamic acid dipeptide repeats protein	Regulates retinoic acid signaling, which is crucial for embryonic development	Neuronal development	[8]
*SLC39A8*	Solute carrier family 39, member 8	Metal ion transporter, involved in the cellular uptake of manganese (Mn) in the brain	Neuronal development	[8]
*SP4*	Specificity protein 4	Encodes a transcription factor that interacts with the promoter region of various genes and modulates their expression; regulates NMDAR abundance	NMDAR formation in the glutamatergic system	[8]
*SRR*	Serine racemase	Regulates NMDAR-dependent synaptic plasticity across neurons	NMDAR function in glutamatergic system dysregulation and synaptic plasticity	[36,37]
*STAG1*	Stromal antigen 1	As a component of the cohesion complex, it regulates chromosomal segregation during anaphase of meiosis	Neuronal maturation	[8]
*ZNF804A*	Zinc finger protein 804A	Encodes a transcription factor and regulates the expression of other genes	Neuronal differentiation	[34]

## Data Availability

No new data were created or analyzed in this study. Data sharing is not applicable to this article.
